# Comparison between Tonifying Kidney Yang and Yin in Treating Segmental Bone Defects Based on the Induced Membrane Technique: An Experimental Study in a Rat Model

**DOI:** 10.1155/2020/6575127

**Published:** 2020-12-25

**Authors:** Zhen Shen, Zehua Chen, Xiaodong Shi, Tao Wang, Minling Huang, Guoqian Chen, Xiangling Ye, Chengzhi Hou, Wengang Liu, Wei Dong, Ying Guo

**Affiliations:** ^1^Department of Orthopaedics, Kunming Municipal Hospital of Traditional Chinese Medicine, Kunming 650011, China; ^2^The Third Affiliated Hospital of Yunnan University of Chinese Medicine, Kunming 650011, China; ^3^The Fifth Clinical Medical College, Guangzhou University of Chinese Medicine, Guangzhou 510095, China; ^4^The First Clinical Medical College, Guangzhou University of Chinese Medicine, Guangzhou 510405, China; ^5^Academy of Chinese Medical Sciences, Beijing 100700, China

## Abstract

Tonifying kidney therapy consisting of tonifying kidney yang and yin is the basic principle of Chinese medicine in treating segmental bone defects (SBDs). Previous studies have demonstrated the presence of the differences between tonifying kidney yang and yin in bone metabolism of osteoporosis and distraction osteogenesis models. However, whether the difference between the two tonifying kidney methods in bone repair for the induced membrane (IM) technique occurs or what is the difference remain unclear. Angiogeneic-osteogenic coupling plays an important role in bone repair and the induced membrane couples angiogenesis with the later osteogenesis during the IM process. This study aimed at investigating the effects of tonifying kidney yang (total flavonoids of Rhizoma Drynariae, TFRD) and yin (plastrum testudinis extract, PTE) on angiogenesis and osteogenesis in the IM-treated SBDs. Rats of 6 mm tibia bone defect model treated with IM were divided into five groups: the control group, the model group, the tonifying kidney yang group (TFRD-treated group), the tonifying kidney yin group (PTE-treated group), and the western medicine group. At 4 weeks after insertion of the polymethylmethacrylate (PMMA), three caudal vertebrae from the tail in each rat were implanted into the 6 mm defect gap. Radiographical, histological, immunohistochemical, and immunofluorescent analyses were performed to assess bone and vessel formation at 4 or 12 weeks after insertion of the PMMA, respectively. Our results revealed that TFRD and PTE were beneficial to both angiogenesis and osteogenesis. TFRD exerted a better effect on angiogenesis than PTE and achieved a better result in stage 1 rather than in stage 2 of IM, whereas PTE was superior to TFRD in osteogenesis and achieved a better result in stage 2 instead of stage 1. Collectively, these findings elucidated the beneficial effects of tonifying kidney yang and yin on angiogenesis and osteogenesis of SBD repair during the IM process, as well as the difference that tonifying kidney yang surpasses tonifying kidney yin in angiogenesis while tonifying kidney yin outperforms tonifying kidney yang in osteogenesis, which suggests that the combination between the application of tonifying kidney yang method in stage 1 of IM and tonifying kidney yin method in stage 2 may achieve better repair efficiency.

## 1. Introduction

Although modern clinical technology is continuously advanced, the management of segmental bone defects (SBDs) remains to be a major ongoing clinical problem in orthopedics. It is reported that SBDs affect more than two million people worldwide every year, which results in significant pain, disability as well as economic and psychosomatic burdens on sufferers because of lengthy treatment duration and high health care costs [1]. It is well known that autologous cancellous bone grafting alone [2] is difficult to cure large segmental bone defects, especially in excess of 4-5 cm [3, 4], which typically requires bone transport via distraction osteogenesis (DO) or bone graft through the induced membrane (IM) technique [5, 6]. In our previous study [7], we have found that IM has advantages over DO and is more suitable and efficient than DO in treating oversized SBDs with the results of relatively faster bone formation rate, shorter treatment duration, and better osteogenesis quality.

The IM technique, also known as the Masquelet procedure, is composed of a two-stage procedure in the management of SBDs [8, 9]. In the first stage, the defect site is stabilized with external or internal fixation, and a cement spacer made of polymethylmethacrylate (PMMA) is inserted into the bone defect gap. After soft-tissue closure, over a period of 2–6 weeks, a membrane that encapsulates the PMMA spacer will be induced and formed by the foreign-body reaction to the PMMA bone cement. In the second stage, the induced membrane is opened, the PMMA spacer is removed, and the resulting cavity is filled with autologous bone. The duration time from inserting the PMMA to the formation of the induced membrane is defined as the first stage of IM and the duration time from removal of PMMA spacer and implanting the autologous bone to bone defect repair is defined as the second stage of IM. Since the concept of IM was introduced by the French surgeon Masquelet [8] in 2000, IM has gained great popularity worldwide [10, 11] and has been extensively studied for the treatment of SBDs in clinical practice [12–14]. Various types of clinical reports including prospective [15] or retrospective studies [16, 17] and meta-analyses [18, 19] indicated that the IM technique was effective in treating SBDs. However, it is undeniable that lengthy treatment duration and subsequent complication rates remain a major problem. Worse still, lack of pharmacotherapies to accelerate bone repair in the defect gaps and to permit earlier weight-bearing movement restrict the wide application of IM technique in clinical practice.

Fortunately, tonifying kidney herbs can be used as an effective therapeutic strategy in promoting bone formation and repair [20]. As is well known, tonifying kidney method is the basic principle of Chinese medicine in treating fractures. It is widely considered that tonifying kidney can produce kidney essence and contribute to bone formation. Meanwhile, tonifying kidney can also turn into blood. The therapeutic strategy of tonifying kidney to reinforce bones actually has the meaning of tonifying kidney to enrich the kidney essence that would transform into blood to nourish the bone. Previous studies showed that tonifying kidney treatment could improve bone repair and healing mainly through facilitating osteogenesis of osteolineage cells [21] or inhibiting the osteoclast bone resorption [22]. But of note, skeletal development and repair occur in close spatial and temporal association with angiogenesis [23, 24]. A strong link between the skeletal vascular system and bone tissue has been confirmed during bone development, homeostasis, and even pathology [25–27]. Growing evidence has demonstrated that there is a close interaction between osteogenesis and angiogenesis that is called the angiogenic-osteogenic coupling [28–30]. Since angiogenic-osteogenic coupling plays an important role in bone development and repair, is there a possibility that the mechanism of tonifying kidney-promoted bone formation involves the dual effects of tonifying kidney method on angiogenesis and osteogenesis? Additionally, it is well known that tonifying kidney methods are divided into tonifying kidney yin and tonifying kidney yang. In the previous study, we found that there were differences in the mechanism of bone metabolism between kidney yang and kidney yin tonifying methods [31]. Moreover, the two methods of tonifying kidney also exerted different effects on angiogenesis and osteogenesis during the process of DO technique [32].

Therefore, the aim of the study was to investigate the effects of tonifying kidney on the IM-treated SBDs by dividing into tonifying kidney yin and tonifying kidney yang, from the perspective of “kidney-blood-bone,” which not only reflects the theoretical and therapeutic basis of traditional Chinese medicine in promoting the angiogenic-osteogenic coupling, but also further deepens the understanding of kidney-tonifying treatment.

## 2. Materials and Methods

### 2.1. Regents and Chemicals

Total flavonoids of Rhizoma Drynariae (TFRD) were purchased from Beijing Qihuang Pharmaceutical Manufacturing Co., Ltd. (National Medicine Permit No. Z20030007, the content of TFRD ≥ 80%). Plastrum testudinis was purchased from the first affiliated hospital of Guangzhou University of Chinese Medicine and Plastrum testudinis extracts (PTE) were obtained according to a method established previously [22], with minor modifications. Briefly, Plastrum testudinis was extracted stepwise with solvent of ethyl acetate and water, and then solvents were recovered to obtain ethyl acetate (PTE) that was dissolved in dimethyl sulfoxide. Ossotide tablets (OT) were purchased from Anhui Hongye Pharmaceutical Manufacturing Co., Ltd. (National Medicine Permit number Z20030007). Reagents associated with hematoxylin and eosin (H&E) and Masson's trichrome (Masson's) analyses were obtained from Google Biotechnology Limited Company (Wuhan, China). Anti-CD31 (1 : 200, ab64543), anti-BMP2 (1 : 200, ab14933), and anti-OPN (1 : 200, ab8448) antibodies were purchased from Abcam (USA). Anti-OCN (1 : 100, sc30045) and the HRP-conjugated secondary antibodies were obtained from Santa Cruz (USA).

### 2.2. Experimental Animals

Sixty adult male rats of similar age (10–12 weeks) and weighing (250–270 g) were purchased from the Experimental Animal Center of Guangzhou University of Chinese Medicine, raised in a specific pathogen-free (SPF) laboratory at a temperature of 24°C and a light and dark period of 12 : 12 h, and fed a standard diet. This animal experiment was approved by the Institutional Animal Ethics Committee of the First Affiliated Hospital of Guangzhou University of Chinese Medicine (ethical approval number TCMF1-2018002).

### 2.3. Experimental Model

**Figure 1:**
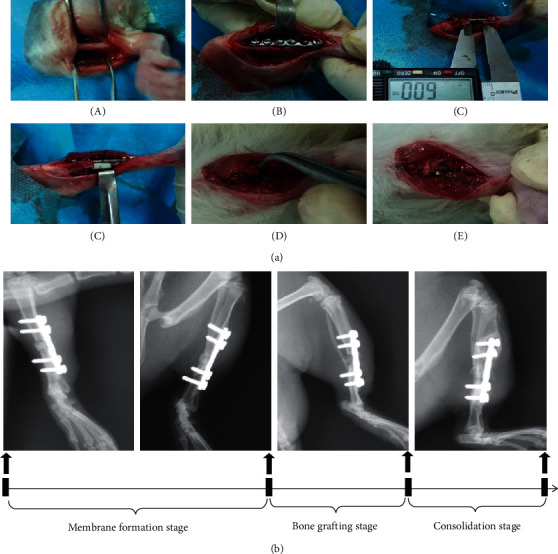
Animal model of the induced membrane (IM) in rats. (a) Flowcharts of surgical procedures of IM model by the internal plate. (A) Tibia exposure; (B) plate fixation; (C) osteotomy; (D) bone cement implantation; (E) induced membrane formation; and (F) bone grafting. (b) Protocols of IM shown by X-ray.

Two weeks after acclimatization to the housing environment, the IM-treated SBD mode was established according to our previous study [7]. Briefly, after intraperitoneal injection of pentobarbital anesthesia, a longitudinal 1 cm incision was made in the skin distal to the tibia crest to expose the bone (Figure 1(A)). Then, a custom-made stainless-steel miniplate was applied to the anterior aspect of the tibia shaft and secured with cortical screws (plate: 15 mm long, 6-hole, screws: 2.0 mm diameter, Baokang, Zhangjiagang, China) (Figure 1(B)). A Gigli saw (Baokang, Zhangjiagang City, Jiangsu Province, China) was used to create a 6 mm long middiaphyseal defect on the right tibia (Figure 1(C)). Following the construction of the above-mentioned SBDs, bone defects of tibias were firstly filled with the polymethylmethacrylate (PMMA) spacers (Figure 1(D)). After flushing the operation area with 0.9% normal saline, the muscle, deep fascia, and skin were sutured layer by layer. Four weeks after the first phase of the operation, a membrane was formed (Figure 1(E)) and, subsequently, removal of the spacer and implantation of autologous corticocancellous bone was performed (Figure 1(F)). Of note, autologous corticocancellous bone was harvested from the same vertebrae of the tail in each rat and soft tissue covering the surface of vertebrae was completely removed before crushing the vertebrae under sterile conditions. After the operation, rats were allowed to eat and drink ad libitum. Antibiotic (amoxicillin 1.5 mg/100 g weight) and buprenorphine (1.0 mg/kg weight) were administered intraperitoneally for the following 3 days, respectively.

### 2.4. Grouping and Administration

The rats were randomly divided into five groups: the tonifying kidney yang group, the tonifying kidney yin group, the western medicine group, the model group, and the control group, with twelve rats in each group. The dose selections of TFRD, PTE, and OT were based on the previous studies [22, 32, 33]. Rats in the tonifying kidney yang group were administered orally with TFRD at a dose of 75 mg/kg body weight/day and rats of the tonifying kidney yin group were orally fed with PTE at a dose of 30 mg/kg body weight/day. The western medicine group received oral OT at a dose of 0.58 mg/kg body weight/day, whereas the model and control groups were subjected to the administration of an equal amount of vehicle. On the first day after modeling, intragastric administration was started once a day for 12 weeks.

### 2.5. X-Ray Analysis

The X-ray photographs were taken at 12 weeks after insertion of the PMMA to show the bone repair (*n* = 3 per group). The X-ray pictures were assessed by three independent orthopedic surgeons who were blind to the treatments and groups. The Lane–Sandhu radiographic scoring method described by Lane and Sandhu [34] was used to score the bone repair, which is shown in Table 1. The following three aspects including bone formation, fracture line healing, and remodeling were evaluated, respectively. All tests were repeated with three specimens.

### 2.6. Micro-CT Analysis

Following X-ray examination, tibias were immediately collected and fixed in 4% paraformaldehyde for approximately 48 hours. Then, samples (*n* = 3 per group) were scanned with micro-CT (SkyScan 1076, Kontich, Belgium) at a resolution of 20 *μ*m (70 kV and 130 *μ*A radiation source with 0.5 mm aluminum filter). The bone tissue volume/total tissue volume (BV/TV) within the defect gaps was analyzed by Scanco software. All tests were repeated with three specimens.

### 2.7. Angiography Analysis

The three rats per group at 4 weeks after insertion of the PMMA were euthanized and perfused with Microfil (Microfil MV-122, Flow Tech; Carver, MA, USA). Briefly, the rib cage was opened after anesthetization, the descending aorta was clamped, and the inferior vena cava was incised. Subsequently, the vasculature was flushed with 0.9% normal saline containing heparin sodium (100 U/mL) and 20 ml of Microfil were, respectively, perfused into the left ventricle with an angiocatheter. Subsequently, the rats were stored at 4°C overnight to ensure polymerization of the contrast agent, after which the tibias were dissected, fixed in 4% paraformaldehyde for 48 hours, decalcified in 10% EDTA for four weeks, and then imaged by micro-CT.

### 2.8. Histological, Immunohistochemical, and Immunofluorescent Analyses

The membrane specimens were taken at 4 and 12 weeks after insertion of the PMMA, and the tibia specimens were taken at 12 weeks after insertion of the PMMA, respectively, followed by fixation in 4% paraformaldehyde for 48 hours. Tibia specimens were decalcified in 10% EDTA for 4 weeks, dehydrated through graded ethanol of increasing concentration, and then embedded in paraffin. Subsequently, all the specimens were cut into 5 *μ*m thick longitudinally oriented sections, of which some were processed for H&E and Masson's staining and others were for immunohistochemical and immunofluorescent staining. For immunohistochemical staining, in brief, the sections embedded in paraffin were dewaxed, rehydrated, and treated with antigen retrieval. Then, the sections were incubated with the primary antibodies to rabbit CD31 (Abcam, UK 1 : 200, ab64543) and BMP2 (Abcam, UK 1 : 200, ab14933) overnight at 4°C. Subsequently, the sections were incubated with the HRP-conjugated secondary antibody (Santa Cruz, USA), followed by counterstaining with hematoxylin. For immunofluorescent staining, briefly, some sections were antigen retrieved, incubated with anti-OPN (1 : 200, ab8448) and anti-OCN (1 : 100, sc30045) primary antibodies, and then stained with secondary antibodies conjugated with fluorescence at room temperature for 1 h. Nuclei were stained with DAPI. Images were acquired with a Leica DMI6000B fluorescence microscope (Solms, Germany) and the positive stained cell numbers and area were quantified using Image J software (5 random visual fields per section, 3 sections per staining, and 3 rats per time point). As shown in Table 2, the bone reconstruction level of the bone graft area was evaluated according to the Huddleston grading scale [35].

### 2.9. Statistical Analysis

GraphPad Prism 6.01 software (La Jolla, CA, USA) was used to analyze the experimental data. All data were presented as means ± standard deviations. Differences among groups were assessed by one-way analysis of variance (ANOVA). *p* < 0.05 was considered to be statistically significant.

## 3. Results

### 3.1. X-Ray Evaluation

**Figure 2:**
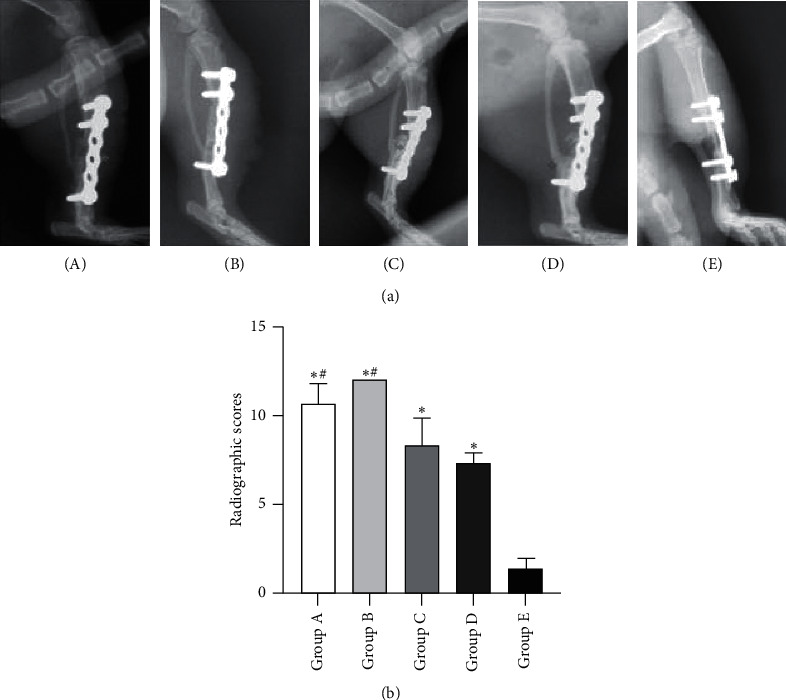
Radiological evaluation of bone repair. (a) Representative radiographs of bone repair of the five groups at 12 weeks after insertion of the PMMA. (b) Quantitative analysis of radiographic scores. (A) Tonifying kidney yang group; (B) tonifying kidney yin group; (C) western medicine group; (D) model group; and (E) control group. ^*∗*^*p* < 0.05, compared with the control group; ^#^*p* < 0.05, compared with the model group.

As presented in Figure 2(a), more newly formed calluses inside the defect gaps were observed in the other four groups than in the control group. Compared with the model group, tonifying kidney yang, tonifying kidney yin, and the western medicine treatments resulted in remarkable increases in bone formation inside the defect gaps. Moreover, the quantitative analysis indicated that the radiologic score of the control group was markedly lower than those of the other four groups and the radiologic score of the model group was obviously lower than those of the tonifying kidney yang, the tonifying kidney yin, and the western medicine groups (Figure 2(b), Table S1). But of note, the result of osteogenesis in the tonifying kidney yin group was better than that in the tonifying kidney yang group, with the manifestation of completely remodeled callus and well recanalized medullary cavity.

### 3.2. Micro-CT Evaluation

**Figure 3:**
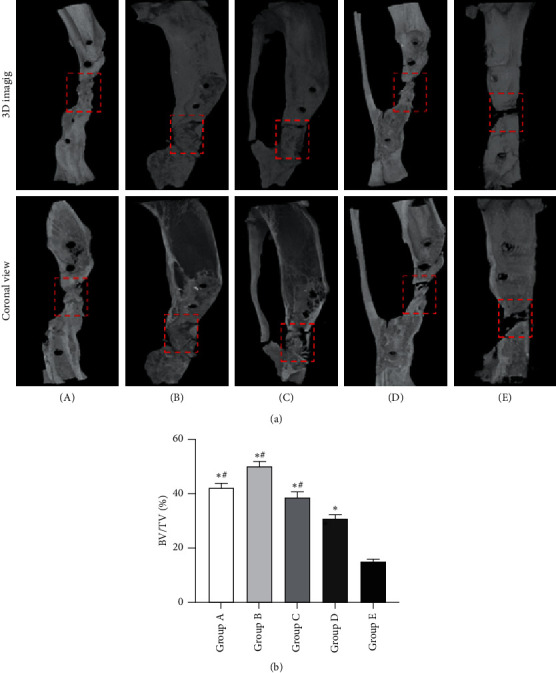
Representative micro-CT images of bone repair. (a) Three-dimensional (top panel) and two-dimensional (lower panel) reconstructed images of bone defects at 12 weeks after insertion of the PMMA. Red dotted boxes indicate the region of interest (ROI), representing bone defect areas. (b) Quantification of bone tissue volume/total tissue volume and (BV/TV) inside defect regions. (A) Tonifying kidney yang group; (B) tonifying kidney yin group; (C) western medicine group; (D) model group; and (E) control group. ^*∗*^*p* < 0.05, compared with the control group; ^#^*p* < 0.05, compared with the model group.

Similar to the manifestation of X-ray photography, the results of Micro-CT showed that tonifying kidney yang and yin groups achieved complete bony union, and the western group achieved partial bony union, while no obvious radiographical defect bridging occurred in the model and control groups (Figure 3(a)). Additionally, BV/TV of all the groups almost showed the same pattern as the radiologic scores. Namely, BV/TV of the tonifying kidney yang, tonifying kidney yin, and western medicine groups were higher than those of the model and control groups. Furthermore, BV/TV of tonifying kidney yin group was higher than that of tonifying kidney yang group (Figure 3(b), Table S2).

### 3.3. Angiography Evaluation

**Figure 4:**
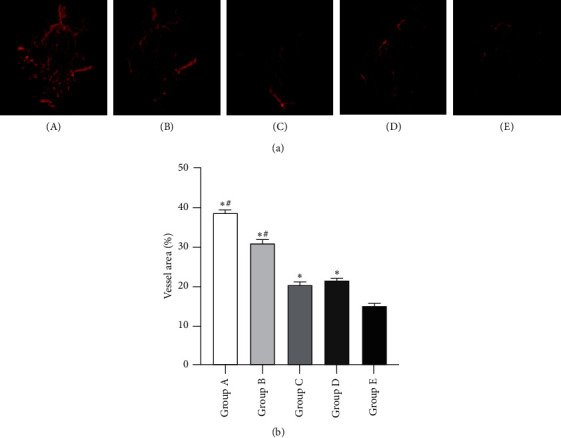
Evaluation of angiogenesis in the induced membrane at 4 weeks after insertion of the PMMA. (a) Representative angiographs of the induced membrane in the five groups. (b) Quantification of vessel area/total membrane area around defect regions. (A) Tonifying kidney yang group; (B) tonifying kidney yin group; (C) western medicine group; (D) model group; and (E) control group. ^*∗*^*p* < 0.05, compared with the control group; ^#^*p* < 0.05, compared with the model group.

As shown in Figure 4(a), the angiography analysis demonstrated that more vessels were observed in the tonifying kidney yang, the tonifying kidney yin, the western medicine, and the model groups than in the control group. Moreover, there were more vessels in the tonifying kidney yang and yin groups than in the model groups, but no significant difference emerged between the western medicine group and the model group (Figure 4(b), Table S3). More importantly, unlike the pattern of osteogenesis revealed by X-ray (Figure 2) and Micro-CT (Figure 3), the tonifying kidney yang method resulted in more formed vessels than the tonifying kidney yin method, while the tonifying kidney yin method contributed to more formed bone than tonifying kidney yang method.

### 3.4. Histological Evaluation of Bone

**Figure 5:**
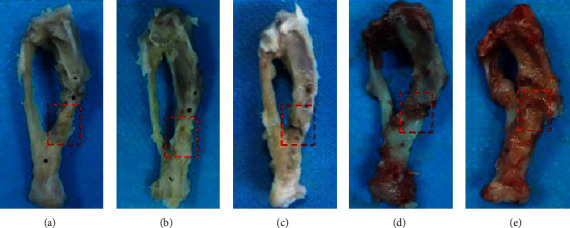
Overview of representative samples harvested from the five groups at 12 weeks after insertion of the PMMA. Red dashed lines outline the defect regions. (a) Tonifying kidney yang group; (b) tonifying kidney yin group; (c) western medicine group; (d) model group; and (e) control group.

**Figure 6:**
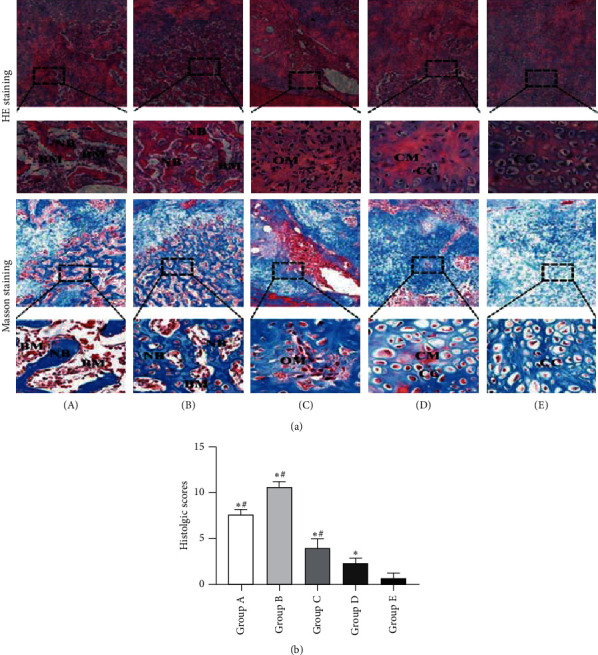
Histological analysis of newly formed tissues within the defect regions at 12 weeks after insertion of the PMMA. (a) Representative HE and Masson's staining images of samples harvested from the five groups. The black boxes represent a higher magnification view of bone defect slices. Original magnification, 40×; higher magnification, 100×. (b) Quantitative analysis of histologic scores. (A) Tonifying kidney yang group; (B) tonifying kidney yin group; (C) western medicine group; (D) model group; and (E) control group. NB: newly formed bone; BM: bone marrow; OM: osteoid matrix; CM: chondroid matrix; CC: chondrocyte. ^*∗*^*p* < 0.05, compared with the control group; ^#^*p* < 0.05, compared with the model group.

Gross views of bone revealed that complete bony union inside defect regions have been achieved in tonifying kidney yang and yin groups at 12 weeks after insertion of the PMMA, while partial bony defect bridging occurred in the western medicine group and almost soft-tissue union was observed in the model and control groups (Figure 5), which is further confirmed by the decalcified sections stained with HE and Masson's. As shown by HE and Masson's staining (Figure 6(a)), tonifying kidney yang and yin groups have experienced the complete defect healing and had the best bone connection and integration with both newly formed bone tissue (NB) bridging the defects and bone marrow (BM) filling up the defect gap, while moderate immature new bone formation with amounts of osteoid matrix appeared inside the defect regions in the western group and only chondroid matrix accompanied with fibrous connective tissues filled in the most area of the defect in the model and control groups. Quantitative analysis of histologic scores was consistent with the results of radiologic scores and BV/TV, which indicated that higher scores were found in tonifying kidney yang and yin groups than in the other three groups and tonifying kidney yin group had the highest score in all the five groups (Figure 6(b), Table S4).

### 3.5. Histological Evaluation of the Membrane

**Figure 7:**
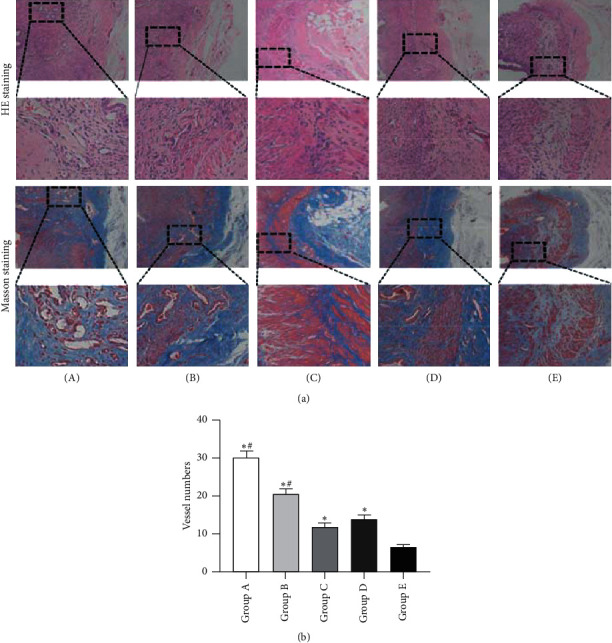
Histological analysis of newly formed tissues within the defect regions at 4 weeks after insertion of the PMMA. (a) Representative HE and Masson's staining images of samples harvested from the five groups. The black boxes represent a higher magnification view of bone defect slices. Original magnification, 40×; higher magnification, 100×. (b) Quantification of newly formed vessels at 4 weeks after insertion of the PMMA (5 random visual fields per section, 3 sections per staining, and 6 sections per rat). (A) Tonifying kidney yang group; (B) tonifying kidney yin group; (C) western medicine group; (D) model group; and (E) control group. ^*∗*^*p* < 0.05, compared with the control group; ^#^*p* < 0.05, compared with the model group.

In line with the result of angiogenesis shown by angiography analysis, the histological analysis further demonstrated that there were more vessels in the four groups in comparison with the control group. Additionally, more vessels were found in tonifying kidney yang and yin groups than in the other three groups and tonifying kidney yang group had the most vessels in all the five groups (Figure 7, Table S5).

### 3.6. Immunohistochemical Evaluation of Membrane

**Figure 8:**
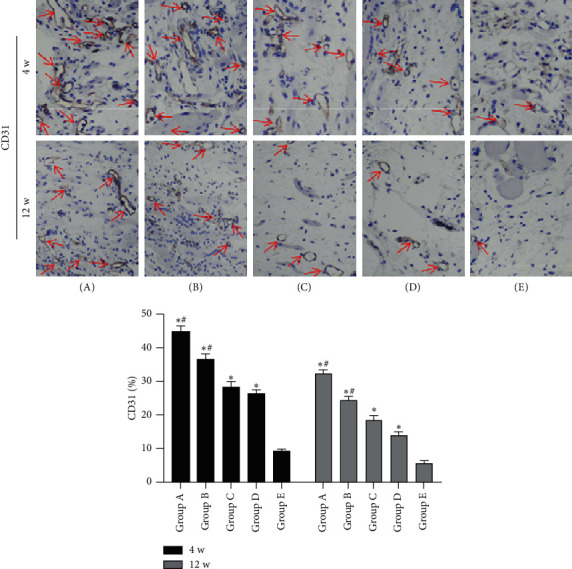
Immunohistochemical results of CD31 in induced membranes around the defect regions of the five groups at 4 and 12 weeks after insertion of the PMMA, respectively. Red arrows indicate the CD31-positive cells (5 random visual fields per section, 3 sections per rat). Original magnification, 200×. (A) Tonifying kidney yang group; (B) tonifying kidney yin group; (C) western medicine group; (D) model group; and (E) control group. ^*∗*^*p* < 0.05, compared with the control group; ^#^*p* < 0.05, compared with the model group.

**Figure 9:**
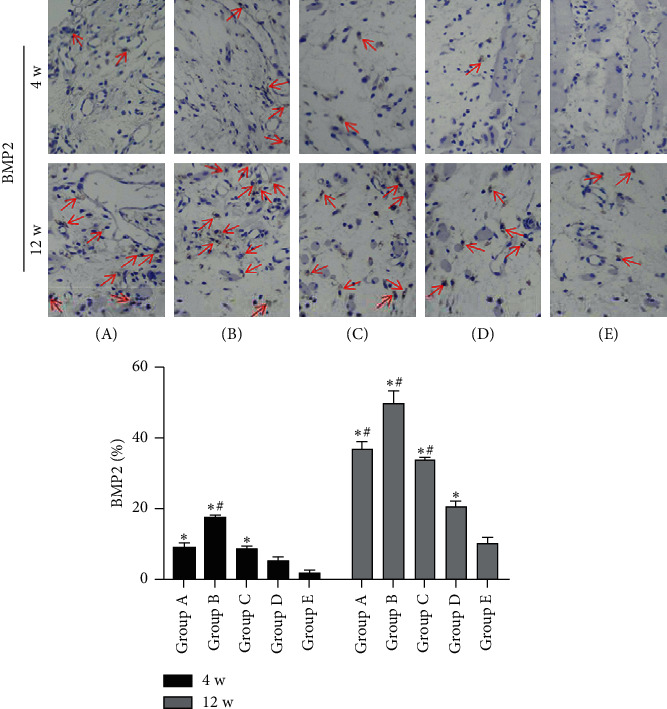
Immunohistochemical results of BMP2 in induced membranes around the defect regions of the five groups at 4 and 8 weeks after insertion of the PMMA, respectively. Red arrows indicate the CD31-positive cells (5 random visual fields per section, 3 sections per rat). Original magnification, 200×. (A) Tonifying kidney yang group; (B) tonifying kidney yin group; (C) western medicine group; (D) model group; and (E) control group. ^*∗*^*p* < 0.05, compared with the control group; ^#^*p* < 0.05, compared with the model group.

Generally, the results of immunohistochemistry staining and quantitative analysis indicated that the percentages of CD31-positive cells in the induced membranes decreased over time (Figure 8, Table S6), while the percentages of BMP2-positive cells increased with time (Figure 9, Table S7). As demonstrated in Figure 8, the percentages of CD31-positive cells at 4 weeks after insertion of the PMMA were higher than those at 12 weeks after insertion of the PMMA. Compared with the other three groups, tonifying kidney yang and yin groups could ameliorate the decline in the percentage of CD31-positive cells and subsequently had higher percentages of CD31-positive cells. But of note, no matter at 4 weeks or 12 weeks after insertion of the PMMA, the percentages of CD31-positive cells in tonifying kidney yang groups were higher than those in tonifying kidney yin groups.

By contrast, the percentages of BMP2-positive cells at 4 weeks after insertion of the PMMA were lower than those at 12 weeks after insertion of the PMMA. Compared with the other three groups, tonifying kidney yang and yin groups could amplify the ascent in the percentage of BMP2-positive cells and subsequently achieved higher percentages of BMP2-positive cells. But it is of note that the percentages of BMP2-positive cells in tonifying kidney yang groups were lower than those in tonifying kidney yin groups at 4 and 12 weeks after insertion of the PMMA, respectively (Figure 9).

### 3.7. Immunofluorescent Evaluation of Bone

**Figure 10:**
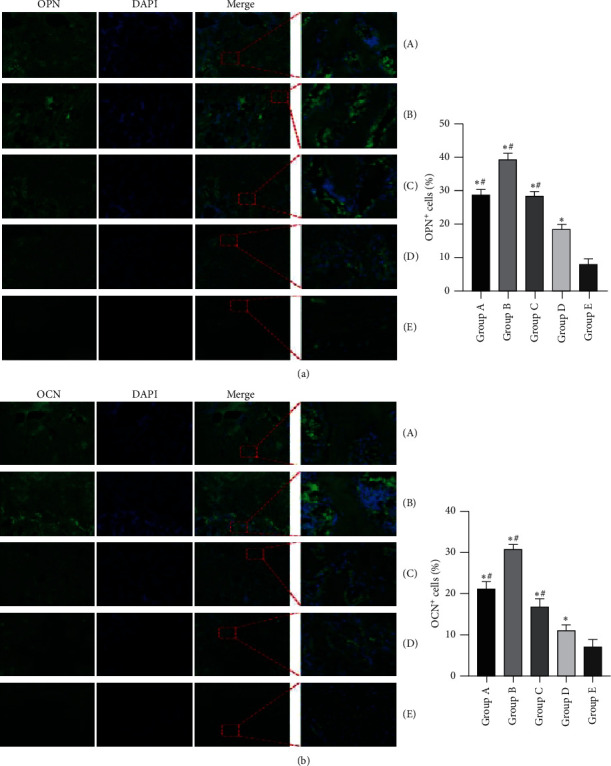
Immunostaining results of OPN and OCN in newly formed tissues within the defect regions of the five groups at 12 weeks after insertion of the PMMA. (a) Representative images and quantification of OPN+ cells in the newly formed tissues. (b) Representative images and quantification of OCN+ cells in the newly formed tissues. Original magnification, 40×; higher magnification, 200×. (A) Tonifying kidney yang group; (B) tonifying kidney yin group; (C) western medicine group; (D) model group; and (E) control group. ^*∗*^*p* < 0.05, compared with the control group; ^#^*p* < 0.05, compared with the model group.

As shown in Figure 10, the medium-term osteogenic marker OPN (Figure 10(a)) and the late-term osteogenic marker OCN (Figure 10(b)) were detected inside the defect regions at 12 weeks after insertion of the PMMA. More importantly, the expressions of OPN and OCN detected by the immunofluorescent assays further verified the radiographical and histological analyses, which demonstrated that the percentages of OPN- and OCN-positive cells in tonifying kidney yang and yin groups were higher than those in the model and control groups and tonifying kidney yin group had a higher percentage of OPN- and OCN-positive cells than tonifying kidney yang group (Figure 10, Table S8).

## 4. Discussion

Bone is a highly vascularized tissue that has an extensive network of blood vessels [36]. Skeletal blood vessels not only act as a transport conduit system that enables the supply of different cell types with efficient and optimal delivery of oxygen and nutrients to the bone injured sites [37] but also provide a true niche for pericytic mesenchymal stem cell- (MSC-) like cells and become a source of MSCs or osteoprogenitors accompanied with hormones, growth factors, calcium, and phosphate for matrix mineralization [38–41], which indicates that bone vasculature is essential for bone formation and repair. More importantly, osteogenesis is frequently coupled with angiogenesis during bone modeling and remodeling. Large numbers of studies have demonstrated that the angiogenic-osteogenic coupling involves bone development, growth, repair, and even aging [28, 42–44]. Various kinds of skeletal cells including osteoprogenitors, osteoblasts, and chondrocytes secrete angiogenic growth factors that regulate endothelial cell behavior and subsequently promote vessel growth, whereas endothelial cells produce angiocrine factors and osteogenic signals that act on bone-forming cells [37].

For the IM technique, angiogenesis has been confirmed during the course of IM treatment, especially in the first phase, namely, the stage of inducing membrane formation [45]. Further understanding of the membrane development as well as biological properties will help to optimize the current procedures and subsequently improve repair of SBDs. As described previously [46, 47], membranous tissue enriched in capillaries can be induced and generated due to the foreign-body reaction to the PMMA in the first stage of IM and is vital to the following bone integration and regeneration after implanting autologous bone in the second stage, which suggests that angiogenic-osteogenic coupling also plays an important role in the IM technique [48]. For one thing, as a richly vascularized tissue with numerous small capillaries, the IM not only secretes vascular growth factors and establishes a blood supply for the subsequent SBD repair [49], but also revascularizes the bone graft and prevents resorption of the graft [9, 50]. For another, the IM is possessed of the well-characterized osteogenesis-improving capabilities by secreting osteoinductive growth factors [49, 51]. However, the properties of angiogenesis and osteogenesis in the IM displayed temporal specificity, which means the angiogenic and osteogenic characteristics of the IM is not invariable but changeable with time.

As is well known, the IM is essentially a kind of biomembrane that pertains to be foreign-body granulation tissue formed around the PMMA spacer [49, 51]. Initially, the membrane appeared to be a synovium-like epithelium with the characteristics of a highly vascularized structure that was responsible for the secretion of vascular and osteoinductive factors [50]. Interestingly, with the gradual maturation, the IM gets thicker and resembles periosteum with the cellular composition and molecular profile facilitating SBD repair [46]. However, as time went on, differences emerged in morphology and growth factor expressions between the IM and periosteum. For the IM, the thickness and vascular density decreased with time [52] and the IM gradually turned into an organized pseudosynovial membrane that was still rich in osteogenic factors [50], which was also confirmed by Gindraux et al. [53] who reported that the IM could retain its powerful osteogenic properties over time. Further speaking, the temporal specificity of angiogenic and osteogenic properties of the IM manifests the differences in angiogenesis and osteogenesis between the first and the second stage of IM. Large amounts of newly formed vessels predominantly occurred in the first stage, while the second stage was characterized by bone formation, integration, and regeneration. Due to the angiogenic-osteogenic coupling, the second-stage procedure tends to be subjected to the activities of the membrane formed in the first stage of the IM. It is of interest that some researchers [51, 52] suggested that the optimal time for performing the second-stage surgery should be within 4 weeks after the PMMA implantation, while others [53] reported that the second stage of the IM technique could be performed later. The former view was based on the decline in the ability of angiogenesis with the decreasing growth factor expressions and subsequent reduced osteogenic capacity over time, whereas the latter view was attributed to the fact that the IM could maintain its powerful osteogenic properties with time, which demonstrated that divergence of the optimal time for performing the second-stage surgery arose from the differences in angiogenesis and osteogenesis of the IM at different stages.

In this study, we found that the quality and quantity of bone tissue within the defect regions improved significantly after TFRD or PTE treatment through the radiological and histological analyses, which revealed that tonifying kidney therapies including tonifying kidney yang and yin treatments could exert positive effects on bone formation and repair, similar or even superior to ossotide tablets. Meanwhile, Microfil perfusion and histological results also revealed more vessels within the defect regions in tonifying kidney yang and yin groups than in the other three groups, which demonstrated that tonifying kidney yang and yin methods could promote both angiogenesis and osteogenesis to varying degrees. However, it is of note that tonifying kidney yang was better than tonifying kidney yin in promoting angiogenesis, whereas tonifying kidney yin was superior to tonifying kidney yang in facilitating osteogenesis, which coincided with our previous findings in the DO model [32]. Moreover, the CD31 and BMP2 immunostaining results further confirmed the findings above and indicated that the angiogenic activity of the induced membrane declined, but the osteogenic properties increased and kept a high level over time. These findings were consistent with the previous reports, in which expressions of proangiogenic growth factors including VEGF and TGF-*β* were detected in the membranes as early as the second week and gradually decreased during the following weeks [50] and [54], whereas the concentration of BMP2 was low from two to four weeks after induction, but thereafter increased to a higher level from four to six weeks after induction [55] and even maintained the high level later [53].

In order to further determine the higher property of the second stage of IM, the immunofluorescent analyses were performed to detect the expressions of osteogenic factors including OPN and OCN in the newly formed tissues within the bone defect regions. As revealed by the immunofluorescent analyses in the present study, all the five groups could detect the presence of OPN+ and OCN+ cells indicating the occurrence of osteogenic differentiation and bone formation in the second stage of IM. In addition, the expressions of OPN and OCN in tonifying kidney yang and tonifying kidney yin groups were obviously higher than those in the other three groups, which further verified that the second stage was featured by bone formation, integration, and regeneration. Moreover, compared with tonifying kidney yin group, tonifying kidney yang group achieved much higher expressions of OPN and OCN, which demonstrated that tonifying kidney yang outperformed tonifying kidney yin in upregulating the osteogenic factors and promoting bone formation and repair.

## 5. Conclusion

Taken together, both tonifying kidney yang method and tonifying kidney yang method can upregulate the expressions of angiogenic and osteogenic factors in the induced membranes and subsequently promote angiogenesis and osteogenesis, but there are differences in the mechanism between the two. Tonifying kidney yang exerted a better effect on angiogenesis than tonifying kidney yin and achieved the better result in the first stage rather than in the second stage, whereas tonifying kidney yin was superior to tonifying kidney yang in osteogenesis and achieved the better result in the second stage instead of the first stage of the IM technique.

In this study, the effects of two tonifying kidney methods on angiogenesis and osteogenesis were observed at different time stages of the IM technique to investigate the effect of IM on SBDs, which shows innovation to a certain extent. Compared with the single application of tonifying kidney yang or tonifying kidney yin during the whole process, or a certain prescription with dual tonifying efficiency in kidney yang and kidney yin during the whole process, the combination between the application of tonifying kidney yang method in the first stage and the application of tonifying kidney yin method in the second stage is more targeted and achieves better bone repair efficiency, which not only substantially improves our knowledge of the scientific meanings of the theory of kidney governing bones in traditional Chinese medicine, but also provides references for in-depth exploration of the mechanism of tonifying kidney methods in IM as well as practical clinical application.

## Figures and Tables

**Table 1:** Radiographic scoring.

	Points
Bone formation	
No evidence of bone formation	**0**
Bone formation occupying 25% of defect	**1**
Bone formation occupying 50% of defect	**2**
Bone formation occupying 75% of defect	**3**
Full-gap bone formation	**4**

Union	
Full-fracture line	**0**
Partial fracture line	**2**
Absent fracture line	**4**

Remodeling	
No evidence of remodeling	**0**
Remodeling of intramedullary canal	**2**
Full remodeling of cortex	**4**

*Note*. Table 1 is reproduced from Shen et al. [7].

**Table 2:** Histologic scoring.

	Points
Union	
No evidence of union	**0**
Fibrous union	**1**
Osteochondral union	**2**
Bone union	**3**
Complete reorganization of shaft	**4**

Spongiosa	
No osseous cellular activity	**0**
Early apposition of new bone	**1**
Active apposition of new bone	**2**
Reorganizing spongiosa (osteoclasts present)	**3**
Completely reorganized spongiosa	**4**

Cortex	
None	**0**
Early appearance	**1**
Formation under way	**2**
Mostly reorganized	**3**
Completely formed	**4**

## Data Availability

The data used to support the findings of this study are available from the corresponding author upon request.

## Supplementary Materials

Supplementary Materials Table S1: raw data for the X-ray analysis in Figure 2(b). Table S2: raw data for the Micro-CT analysis in Figure 3(b). Table S3: raw data for the angiography analysis in Figure 4(b). Table S4: raw data for the histological analysis in Figure 6(b). Table S5: raw data for the histological analysis in Figure 7(b). Table S6: raw data for the immunohistochemical analysis in Figure 8. Table S7: raw data for the immunohistochemical analysis in Figure 9. Table S8: raw data for the immunohistochemical analysis in Figure 10.
