# Supplementary material for: Comparison between Tonifying Kidney Yang and Yin in Treating Segmental Bone Defects Based on the Induced Membrane Technique: An Experimental Study in a Rat Model
Source: Evid Based Complement Alternat Med. 2020 Dec 25;2020:6575127. doi: 10.1155/2020/6575127 (PMC7781691; doi:10.1155/2020/6575127)
Supplement: Supplementary Materials — Table S1: raw data for the X-ray analysis in Figure 2(b). Table S2: raw data for the Micro-CT analysis in Figure 3(b). Table S3: raw data for the angiography analysis in Figure 4(b). Table S4: raw data for the histological analysis in Figure 6(b). Table S5: raw data for the histological analysis in Figure 7(b). Table S6: raw data for the immunohistochemical analysis in Figure 8. Table S7: raw data for the immunohistochemical analysis in Figure 9. Table S8: raw data for the immunohistochemical analysis in Figure 10. [file 6575127.f1.doc]

Supplementary Material

Table S1. Raw data for the X-ray analysis in Figure 2b.

| **Group** | **N** | **Radiographic score** |
| --- | --- | --- |
| Group A | 3 | 10.667±1.155^*#^ |
| Group B | 3 | 12.000±0.0000^*#^ |
| Group C | 3 | 7.333±1.528^*^ |
| Group D | 3 | 6.667±0.577^*#^ |
| Group E | 3 | 1.667±0.577 |

Group A: tonifying kidney-yang group; B: tonifying kidney-yin group; C: western medicine group; D: model group; E: control group.Data are expressed as mean±SD. *^*^p* < 0.05, compared with the control group;*^#^p* < 0.05, compared with the model group.

Table S2. Raw data for the Micro-CT analysis in Figure 3b.

| **Group** | **N** | **BV/TV(%)** |
| --- | --- | --- |
| Group A | 3 | 42.233±1.701^*#^ |
| Group B | 3 | 49.933±2.259^*#^ |
| Group C | 3 | 38.567±2.120^*#^ |
| Group D | 3 | 30.900±1.277^*^ |
| Group E | 3 | 14.800 ±0.819 |

Group A: tonifying kidney-yang group; B: tonifying kidney-yin group; C: western medicine group; D: model group; E: control group. Data are expressed as mean±SD. *^*^p* < 0.05, compared with the control group; *^#^p* < 0.05, compared with the model group.

Table S3. Raw data for the angiography analysis in Figure 4b.

| **Group** | **N** | **Vessel area(%)** |
| --- | --- | --- |
| Group A | 3 | 38.958±1.567^*#^ |
| Group B | 3 | 31.572±2.211^*#^ |
| Group C | 3 | 20.215±1.223^*^ |
| Group D | 3 | 22.380±0.936^*^ |
| Group E | 3 | 14.643±1.397 |

Group A: tonifying kidney-yang group; B: tonifying kidney-yin group; C: western medicine group; D: model group; E: control group. Data are expressed as mean±SD. *^*^p* < 0.05, compared with the control group; *^#^p* < 0.05, compared with the model group.

Table S4. Raw data for the histological analysis in Figure 6b.

| **Group** | **N** | **Histologic score** |
| --- | --- | --- |
| Group A | 3 | 7.667±0.577^*#^ |
| Group B | 3 | 10.667±0.577^*#^ |
| Group C | 3 | 4.000±1.000^*#^ |
| Group D | 3 | 2.333±0.577^*^ |
| Group E | 3 | 0.667±0.577 |

Group A: tonifying kidney-yang group; B: tonifying kidney-yin group; C: western medicine group; D: model group; E: control group. Data are expressed as mean±SD. *^*^p* < 0.05, compared with the control group; *^#^p* < 0.05, compared with the model group.

Table S5. Raw data for the histological analysis in Figure 7b.

| **Group** | **N** | **Vessel number** |
| --- | --- | --- |
| Group A | 3 | 30.333±1.528^*#^ |
| Group B | 3 | 20.667±1.155^*#^ |
| Group C | 3 | 12.000±1.000^*^ |
| Group D | 3 | 14.000±1.000^*^ |
| Group E | 3 | 6.667±0.577 |

Group A: tonifying kidney-yang group; B: tonifying kidney-yin group; C: western medicine group; D: model group; E: control group. Data are expressed as mean±SD. *^*^p* < 0.05, compared with the control group; *^#^p* < 0.05, compared with the model group.

Table S6. Raw data for the immunohistochemical analysis in Figure 8.

| **Group** | **N** | **CD31(%)** | |
| --- | --- | --- | --- |
|  |  | **4 w** | **12 w** |
| Group A | 3 | 44.667±2.082^*#^ | 32.333±1.155^*#^ |
| Group B | 3 | 36.667±1.528^*#^ | 24.333±1.155^*#^ |
| Group C | 3 | 28.333±1.528^*^ | 18.333±1.528^*^ |
| Group D | 3 | 26.333±1.155^*^ | 14.000±1.000^*^ |
| Group E | 3 | 9.333± 0.577 | 5.667± 0.577 |

Group A: tonifying kidney-yang group; B: tonifying kidney-yin group; C: western medicine group; D: model group; E: control group. Data are expressed as mean±SD. *^*^p* < 0.05, compared with the control group; *^#^p* < 0.05, compared with the model group.

Table S7. Raw data for the immunohistochemical analysis in Figure 9.

| **Group** | **N** | **BMP2(%)** | |
| --- | --- | --- | --- |
|  |  | **4 w** | **12 w** |
| Group A | 3 | 8.933±1.050^*^ | 36.533±2.173^*#^ |
| Group B | 3 | 17.333±0.577^*#^ | 49.867±3.287^*#^ |
| Group C | 3 | 8.433±0.666^*^ | 33.700±0.721^*#^ |
| Group D | 3 | 5.000±1.000 | 20.667±1.155^*^ |
| Group E | 3 | 1.667±0.577 | 9.967±1.762 |

Group A: tonifying kidney-yang group; B: tonifying kidney-yin group; C: western medicine group; D: model group; E: control group. Data are expressed as mean±SD. *^*^p* < 0.05, compared with the control group; *^#^p* < 0.05, compared with the model group.

Table S8. Raw data for the immunohistochemical analysis in Figure 10.

| **Group** | **N** | **OPN(%)** | **OCN(%)** |
| --- | --- | --- | --- |
| Group A | 3 | 28.599±1.587^*#^ | 21.123±1.821^*#^ |
| Group B | 3 | 39.288±1.863^*#^ | 30.817±1.161^*#^ |
| Group C | 3 | 28.362±1.055^*#^ | 16.849±1.845^*#^ |
| Group D | 3 | 18.371±1.334^*^ | 11.197±1.226^*^ |
| Group E | 3 | 7.744±1.665 | 7.057±1.835 |

Group A: tonifying kidney-yang group; B: tonifying kidney-yin group; C: western medicine group; D: model group; E: control group. Data are expressed as mean±SD. *^*^p* < 0.05, compared with the control group; *^#^p* < 0.05, compared with the model group.
